# Impact of Thermal Variation on Egg Hatching and the Life Cycle of *Aedes* (*Protomacleaya*) *terrens* (Diptera: Culicidae) in a Laboratory Environment

**DOI:** 10.3390/life15071038

**Published:** 2025-06-30

**Authors:** Rayane Dias, Manuella Pereira Cerqueira Leite, Guilherme Sanches Corrêa-do-Nascimento, Gabriel Silva Santos, Cecilia Ferreira de Mello, Nathália Menezes de Almeida, Jeronimo Alencar

**Affiliations:** 1 Laboratório de Diptera, Instituto Oswaldo Cruz (Fiocruz), Rio de Janeiro 21040-360, RJ, Brazil; rayanedias@aluno.fiocruz.br (R.D.); manuella.cerqueira@ufrrj.br (M.P.C.L.); cecilia.mello@ioc.fiocruz.br (C.F.d.M.); nathalia.mda@ufrrj.br (N.M.d.A.); 2 Programa de Pós-Graduação em Medicina Tropical, Instituto Oswaldo Cruz (Fiocruz), Rio de Janeiro 21040-360, RJ, Brazil; 3 Programa de Pós-Graduação em Biologia Animal, Universidade Federal Rural do Rio de Janeiro, Seropédica 23890-000, RJ, Brazil; 4 National Institute of the Atlantic Forest (INMA), Santa Teresa 29650-000, ES, Brazil; guisanchescn@gmail.com; 5 Department of Biological Sciences, Federal University of Espírito Santo, Vitória 29075-910, ES, Brazil; gabriel.s.santos@ufes.br

**Keywords:** Aedini, development, behavior, thermal criteria, biological vectors

## Abstract

Evaluating the development process of mosquito species under the influence of temperature is essential for understanding their ecology and geographical distribution, as well as assessing their potential as vectors of pathogens. *Aedes* (*Protomacleaya*) *terrens*, a species recognized for its susceptibility and competence in transmitting the chikungunya virus, serves as a relevant model for research in this context. This study aimed to analyze the influence of temperature on egg hatching and the development cycle of this species to expand knowledge on its biology and implications for public health. During the experiment, 800 eggs were used, collected through 10 ovitraps in a forest remnant located in Uruaçu, Goiás, Brazil. The total number of eggs was divided into four groups, exposed to constant temperatures of 15 ± 2 °C, 20 ± 2 °C, 25 ± 2 °C, and 30 ± 2 °C. After hatching, first-instar larvae were individually separated and monitored daily under controlled conditions until adult emergence. The highest hatching rate occurred at 25 °C, showing an optimal point around 27 °C. Throughout development, temperature significantly reduced the duration of each stage, with the fastest complete cycle at 30 °C, a difference of approximately 10–12 days when compared to 20 °C and approximately 47 days when compared to 25 °C. These results offer valuable insights into the temperature sensitivity of *Ae*. *terrens* across its developmental stages, suggesting that each stage has its own optimal temperature. Thus, small variations in responses to environmental conditions and differentiation between sexes may become more pronounced throughout development. In this sense, temperature can affect not only the development and survival of dipterans but also the capacity for virus transmission, as the pathogen influences the reproduction rate and longevity of the vectors.

## 1. Introduction

The development and population dynamics of mosquitoes are directly influenced by abiotic factors, such as temperature, relative humidity, and the availability of environmental resources. These effects can be observed in terms of development time, fecundity, body size, and longevity [1]. However, despite the well-established dependence of these insects on abiotic conditions, there is still a significant gap in understanding how exactly these factors determine, either positively or negatively, the development of different life cycle stages, depending on each mosquito species. For example, some species are more tolerant of variations in temperature and humidity, while others may be more sensitive to abrupt climate changes, which can affect their geographic distribution and abundance [2,3,4].

It is also important to understand that the relationship with temperature is not always linear, and development tends to be most efficient at specific temperatures, with survival or development reducing beyond the optimal range. Investigating how different mosquito species respond to temperature can enhance our understanding of their ecology, geographic distribution, and potential as pathogen vectors in various regions of the world [2].

*Aedes* (*Protomacleaya*) *terrens* (Walker, 1856) is a species widely distributed in the tropical and subtropical regions of the Americas. It stands out for its ability to adapt to a variety of larval habitats, with a tendency to proliferate in elevated locations—characteristic of its acrodendrophilic behavior. Its immature forms are often found in bamboo cavities, tree holes, and other elevated substrates, where rainwater accumulates, creating a suitable environment for their development. Unlike other mosquito species, such as *Aedes aegypti* (Linnaeus, 1762) and *Aedes albopictus* (Skuse, 1894), which typically utilize larval habitats closer to the ground, *Ae. terrens* tends to favor higher locations. This characteristic reduces the species’ susceptibility to certain environmental disturbances, such as flooding, while simultaneously making it more difficult to detect and control [5,6,7,8,9].

Although *Ae. terrens* exhibits an acrodendrophilic behavior [10], some studies indicate plasticity in its distribution along the ground–canopy gradient [11], which enhances human–mosquito contact and, consequently, may increase the potential for pathogen transmission associated with the species. *Aedes terrens* prefers habitats near primates and is predominantly associated with wild ecosystems [12]. Galindo, Trapido and Carpenter (1951) [13] conducted pioneering research on the dispersal capacity of *A. terrens* in the state of Minas Gerais, Brazil, using mark–release–recapture experiments. The authors found that adult mosquitoes were recaptured 4.3–4.4 km from the release site within just 1–2 days, highlighting the species’ remarkable ability to disperse over short timeframes. As for activity patterns, available evidence suggests that the species is primarily diurnal. Galindo, Carpenter, and Trapido (1955) [14] reported that oviposition tends to occur in tree cavities, with eggs laid approximately 2.5 cm above the water surface.

In a later study, Ribeiro and Walter (2001) [15] investigated egg hatching in *Ae. terrens* using bamboo containers filled with decomposing organic matter. They found that most eggs hatched after the first immersion in water, although a substantial number still hatched following the fifth immersion. These findings point to an adaptive strategy for resisting desiccation, enabling the species to persist in environments with fluctuating water levels. Furthermore, it has been experimentally demonstrated that the species is highly susceptible to and capable of transmitting the chikungunya virus (CHIKV), a mosquito-borne viral disease responsible for significant outbreaks across various regions of the Americas, raising growing public health concerns. Given the vector competence of *Ae. terrens* and its presence in natural habitats, it is crucial to intensify research on its biology, ecology, and behavior. This will enhance our understanding of its interactions with viruses, as well as its natural and urban environments, providing valuable insights for the development of more effective control and prevention strategies.

Evaluating the impact of temperature on egg hatching and the life cycle of *Ae. terrens* is essential for understanding its biology and public health implications. Studying these effects under laboratory conditions provides deeper insights into its ecology, the maintenance of potential arbovirus cycles, and the species’ adaptive responses to environmental changes, ultimately aiding the development of more effective policies.

The present study aimed to assess the influence of temperature on the life cycle of *Ae. terrens* and identify the thermal conditions required for egg hatching and development. Specifically, we examined how this abiotic factor affects hatching rates and the duration of the developmental cycle, testing whether temperature influences these aspects. We also analyzed temperature responses across the various immature stages and determined the optimal temperature for this species.

## 2. Materials and Methods

### 2.1. Ethical Statement

The permanent license for the collection, capture, and transportation of zoological material was granted by the Chico Mendes Institute for Biodiversity Conservation (ICMBio) and the Biodiversity Authorization and Information System (SISBIO) under License No. 84318-4. All team members involved in the collection process were vaccinated against yellow fever.

### 2.2. Study Area

**Figure 1:**
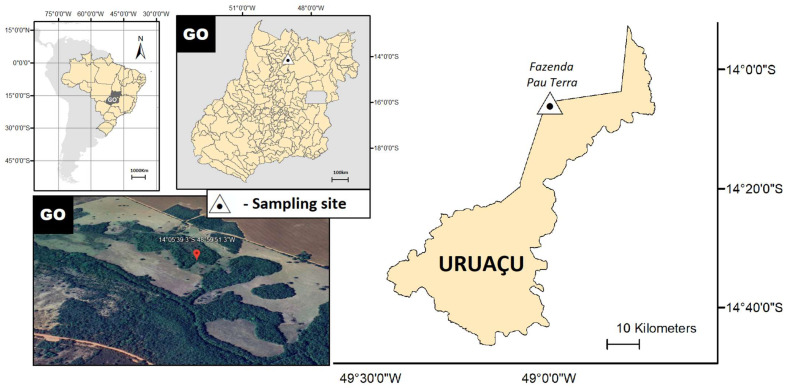
Sampling site at Pau Terra farm, Uruaçu, Goiás, Brazil.

The egg collections were performed at the Pau Terra farm (14°05′38.5″ S; 48°59′51.3″ W), which is located in the municipality of Uruaçu, state of Goiás, Brazil (Figure 1). The landscape of the sampling site consists of a gallery forest that follows small streams, creating a closed corridor over the watercourse. The vegetation is predominantly evergreen, with minimal deciduous vegetation during the dry season, and is surrounded by non-forest vegetation trails on both banks, transitioning abruptly into savannah and field formations [15].

Eggs were collected in December 2023 using 10 ovitraps, each consisted of a matte black plastic container (500 mL capacity, Nutriplan, São Paulo, Brazil) without a lid. Each trap contained 300 mL of water and three rough-surfaced eucalyptus pallets (15 × 3 cm), which were placed vertically inside the trap using clips. These pallets, where females deposited their eggs, were collected after eight days in the field.

### 2.3. Laboratory Experimental Design

In the laboratory, pallets containing eggs were placed in an incubator under controlled conditions of 25 ± 2 °C and 70 ± 10% relative humidity (RH) for 48 h. This period allowed for complete desiccation of the eggs while preserving their viability and ensuring proper embryonic development. After this period, the pallets were individually examined under a stereomicroscope to quantify the eggs. The pallets containing eggs were then immersed in dechlorinated water.

A total of 857 eggs were used, divided into four groups, each placed in a polyethylene container identified by its respective temperature. After hatching, all life cycle stages were monitored daily under constant temperatures of 15 ± 2 °C, 20 ± 2 °C, 25 ± 2 °C, and 30 ± 2 °C. Post-embryonic development occurred in a Biochemical Oxygen Demand (BOD) incubator (Eletrolab, São Paulo, Brazil), under 70 ± 10% RH and a 12:12 light/dark photoperiod.

The egg immersion phase consisted of sequential cycles of hydration and desiccation to induce hatching. For this purpose, the pallets containing eggs were subjected to 18 successive immersions in dechlorinated water, carried out under the specific temperature conditions established in the experiment. After hatching, the larvae were individually transferred to numbered 50 mL plastic cups (Coposul, São Paulo, Brazil) (5 × 5 × 3.5 c) containing dechlorinated water, starting from the first instar. They were fed twice a week with ground fish food at a rate of 0.150 g per 10 mL of dechlorinated water until the fourth instar. During the pupal stage, the containers were stored in adapted cages to allow the emergence of adults under controlled conditions. The presence of exuviae determined the start and end of each life stage.

Species were identified by direct observation of morphological characteristics under an optical microscope and through consultation of species descriptions/diagnoses, using dichotomous keys and references, such as Lane (1953) [16], Consoli and Lourenço-de-Oliveira (1994) [17], and Forattini (2002) [18]. Subsequently, all specimens were deposited in the Entomological Collection at the Oswaldo Cruz Institute, Fiocruz, Rio de Janeiro, Brazil.

### 2.4. Statistical Analyses

To evaluate the effect of temperature on egg hatching rates, a generalized linear model (GLM) with a binomial distribution was fitted using R v.4.4.1 (R Core Team, 2024) [19]. Two competing models were considered: one assuming a linear relationship between egg hatching rate and temperature, and the other assuming a quadratic effect of temperature. These two models inform different developmental responses to temperature. The linear model predicts a continuous increase in the hatching rate with rising temperature, whereas the quadratic model predicts an initial increase up to an optimal temperature, followed by a decline. Model selection was based on the corrected Akaike Information Criterion for small sample sizes (AICc), using the MuMIn package [20]. The model with the lowest AICc value was considered the most plausible, and differences greater than two units (∆AIC > 2) were interpreted as substantial support for one model over another [21].

Model validation considered residual normality, dispersion, and the presence of outliers (Supplementary Material Table S1) and was performed using the DHARMa package [22]. DHARMa enables the validation of simulated residuals based on empirical datasets, facilitating robust validation even with small sample sizes [22].

The influence of temperature on the number of days required to reach adulthood and the differences between sexes and ontogenetic stages were assessed. Competing models were also compared to explain how temperature and sex affect development time to the adult stage from initial immersion. Once the most plausible model was identified, this same model structure was applied separately to each ontogenetic stage to verify how differently each stage was affected by temperature. For the analysis applied to each ontogenetic stage, the transition time between stages was standardized to a mean of 0 and a standard deviation of 1 using a z-score transformation to make the models comparable across different stages.

Finally, to estimate the optimal temperature for mosquito development, the performance of egg hatching and the overall development cycle was projected across a continuous temperature gradient from 20 °C to 30 °C at 0.5 °C intervals.

## 3. Results

During the experimental period, 236 eggs hatched, and a total of 200 larvae reached adulthood, distributed as follows: *Aedes terrens* (*n* = 196; 98%), *Aedes albopictus* (*n* = 2; 1%), and *Haemagogus leucocelaenus* (Dyar, 1925) (*n* = 2; 1%). Due to the low number of individuals from other species, all evaluations were conducted solely for *Ae. terrens*.

### 3.1. Egg Hatching × Temperature

**Figure 2:**
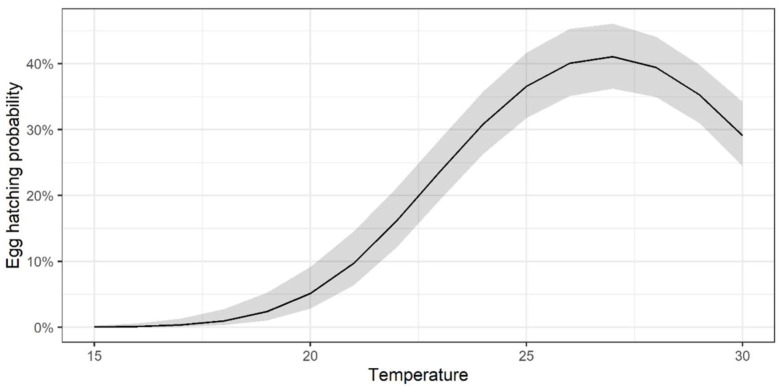
The probability of *Aedes* (*Protomacleaya*) *terrens* egg hatching increases with temperature up to an optimum, then decreases as temperature rises. The figure shows the estimated values (black line) and confidence intervals (gray shading) of the hatching rate (y-axis) as a function of temperature (x-axis) according to the quadratic model. The model demonstrates a maximum hatching probability of 0.41% [min. 0.36–max. 0.461%] achieved at approximately 27 °C.

*Aedes terrens* eggs hatched at three of the four tested temperatures, with the highest hatching rate observed at 25 °C, followed by 30 °C and 20 °C (Table 1). No hatching was recorded at 15 °C, suggesting that this temperature exceeded the lower tolerance limit. These results are supported by the model explaining the probability of egg hatching: the model with a quadratic temperature term (df = 3; AICc = 80.1) performed better than the linear model (df = 2; AICc = 159.7), suggesting an optimal temperature of around 27 °C (Figure 2), with a hatching rate of 0.41% [min. 0.36–max. 0.461%]; SE = 0.105. These findings highlight the significant thermal sensitivity of the *Ae*. *terrens* life cycle, with important implications for species monitoring and control under varying environmental conditions.

### 3.2. Development × Temperature

*Aedes terrens* specimens that hatched at 20 °C, 25 °C, and 30 °C showed total development times (from hatching to adult) of 21.9 (±1.95), 14.6 (±1.63), and 11.6 (±1.81) days, respectively. Development at 30 °C was the fastest, with a duration of approximately 10–12 days shorter than at 20 °C and approximately 4–7 days shorter than at 25 °C (Table 2). Analysis of the different developmental stages revealed that the L4-to-pupa transition had the longest duration across all temperatures. In contrast, the stage with the shortest duration varied: the L2-to-L3 transition at 20 °C and 25 °C and the L3-to-L4 transition at 30 °C (Table 2).

**Figure 3:**
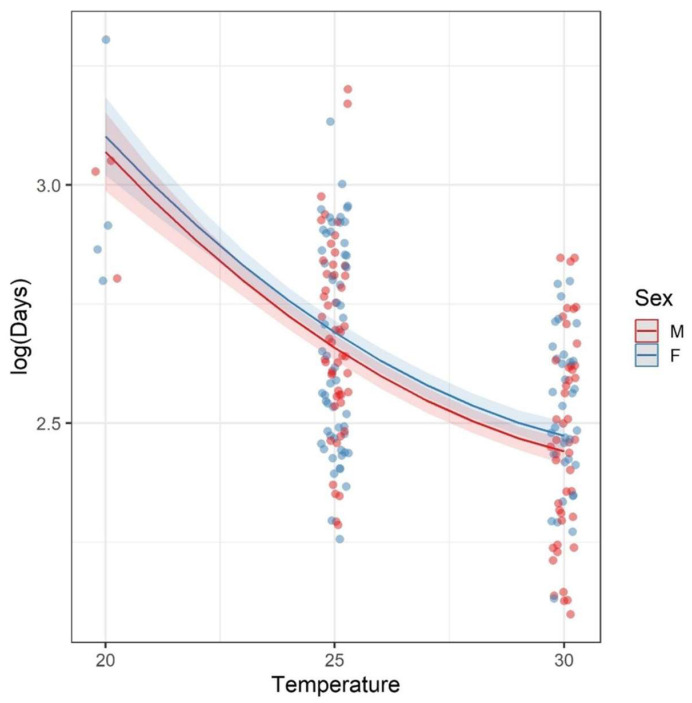
Logarithm of the total number of days for *Aedes* (*Protomacleaya*) *terrens* larvae to reach adulthood (y-axis) as a function of temperature (x-axis) and sex. The curve represents the values estimated by the quadratic model, with the red line corresponding to males and the blue line to females. The shaded areas represent the confidence intervals. The figure shows a slight deceleration in development time as the temperature approaches 30 °C, indicating that although the model suggests the existence of an optimal temperature, it was not identifiable within the tested range.

The most plausible model for explaining development time was again the one including a quadratic temperature term (df = 5; AICc = −308.8). Temperature significantly reduced the time to adulthood from initial immersion (estimated effect of temperature, $\beta_{Temperature}$ = −0.26 ± 0.05 *p* < 0.001; Table 3). Despite relatively close responses, the model also indicated a significant difference between the development times of males and females, with females requiring about one day longer to reach adulthood compared to males (exp ($\beta_{Sex\_M}$) = −0.968; $\beta_{Sex\_M}$ = −0.03 ± 0.015 *p* < 0.001; Table 3). Optimal development values approached the highest temperature tested (30 °C), although a slight deceleration was observed as temperature increased, indicating that while the model suggests the existence of an optimum, it was not identifiable (Figure 3).

**Figure 4:**
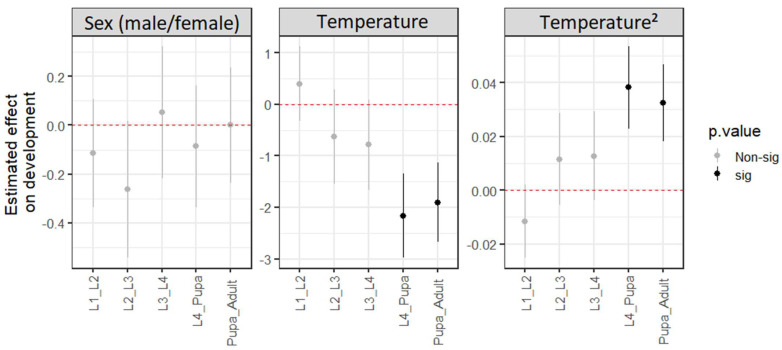
Estimated effects of the variables (difference between females and males on the left, linear temperature effect in the center, and quadratic temperature effect on the right) on the development of *Aedes* (*Protomacleaya*) *terrens* at different stages. The figure shows the mean estimated effect of each variable (sex, temperature, and temperature^2^) and its standard error, as determined by the generalized linear model (GLM) applied to each ontogenetic stage. Significant estimated values for Temperature^2^ suggest that there is an optimal temperature for individual development within or close to the range of temperatures tested in our experiments.

Although temperature affected the overall development of *Ae. terrens* individuals, each immature stage appeared to respond differently to temperature (Figure 4). While some stages seem to be more strongly influenced, others appear less affected. According to the models developed, the strongest temperature effects were detected during the later developmental stages: from L4 to pupa (L4_Pupa; $\beta_{Temperature}$ = −0.566 ± 0.195 *p* = 0.004) and from pupa to adult (Pupa_Adult; $\beta_{Temperature}$ = −0.445 ± 0.155 *p* = 0.004; Figure 4, Table S1). These stages showed a significant contribution from the quadratic temperature term, implying the existence of an optimal temperature close to the temperature range used in our study. Conversely, only the L2–L3 transition showed a significant difference between males and females (L2–L3; $\beta_{Sex}$ = −0.116 ± 0.059 *p* = 0.049; Figure 4, Table S1), which may be associated with the observed overall difference between the sexes in the time required to reach adulthood after immersion. However, there was no evidence of sexual differentiation in the transition time across most developmental stages.

## 4. Discussion

Studying thermal conditions throughout the mosquito life cycle is essential for understanding how environmental factors can influence the ecology and epidemiology of these vectors. By assessing how different temperatures affect development time and the interval to adult emergence, it is possible to gather critical information for controlling pathogen-transmitting mosquitoes. These insights are fundamental for developing effective strategies to combat diseases, such as chikungunya, malaria, dengue, and Zika, which significantly impact public health across various regions worldwide.

In the present study, *Ae. terrens* specimens exhibited varied developmental patterns across different temperatures, affecting both egg hatching and immature stage development, consistent with observations from previous research. Ribeiro et al. (2004) [4] observed that *Culex quinquefasciatus* (Say, 1823) hatched under all temperature conditions tested, achieving a hatching percentage of 97.9%. In contrast, our study revealed no egg hatching at 15 °C, highlighting species-specific thermal sensitivities. Nevertheless, the overall developmental pattern was similar: development accelerated with increasing temperatures, with average durations of 44.3, 19.6, 15.3, and 10.2 days across the temperature range of 15 °C to 30 °C, despite differences in mosquito genera.

While exhibiting a clear optimal temperature for development, *Ae. terrens* also demonstrated adaptability to both lower and higher temperatures. These results suggest potential behavioral changes in response to climatic variations in Brazil, particularly in the central-west region, where the annual average temperature has been rising [23]. Based on these findings, the study area can be considered a favorable environment for increased *Ae. terrens* abundance since higher temperatures generally benefit mosquito vector development, transmission capacity, activity, and reproduction [24]. However, it is important to note that the species can also develop under cooler conditions, allowing populations to migrate to milder regions.

Rodrigues (2004) [25] reported that higher temperatures generally accelerate dipteran development, whereas lower temperatures slow it down. However, different life stages may respond differently to temperature [26]. Specifically for *Ae. terrens*, rising temperatures seem to accelerate development to adulthood, although egg hatching appears negatively affected by higher temperatures. Ecologically, this implies that temperature increases could reduce egg viability but speed up development from immature stages to adulthood.

The impacts of climate change could create more favorable conditions for mosquito-borne pathogen transmission, posing significant public health challenges. In the context of the present study, climatic changes also negatively affected hatching rates. Laboratory observations showed that increasing temperatures above 25 °C primarily benefited larval development rather than egg hatching. In the study area, climatic conditions are generally favorable for mosquito development most of the year, with suitable monthly mean temperatures (21.7 °C to 25.8 °C) and a longer rainy season (9.6 months) than the dry period (2.4 months) [27,28]. Furthermore, meteorological data from nearby stations indicate that average temperatures have been rising over recent decades, bringing them closer to the optimal temperature range (26–28 °C) for the life cycle of most Neotropical mosquitoes [29,30], a range also identified for *Ae.* (*Protomacleaya*) *terrens* egg hatching in this study. Additionally, extreme climatic events, such as heavy rainfall and heat waves, may affect the availability, maintenance, and quality of mosquito larval habitats.

Under laboratory conditions, it is possible to control temperature to assess how different thermal conditions affect the time needed for mosquitoes to complete their life cycle, including immature stage duration and time to adult emergence. It is important to emphasize that different mosquito species may have distinct thermal requirements for egg hatching and life cycle progression. Some species may be more adapted to higher temperatures, while others may prefer cooler conditions.

Calado (2002) [1] found that *Ae. albopictus* egg viability remained above 65% across all evaluated temperatures (15 °C, 20 °C, 25 °C and 30 °C), whereas *Ae. terrens* in the present study showed no hatching at 15 °C, revealing a different thermal tolerance even within the *Aedes* genus. While the total developmental duration followed the expected pattern of decreasing with rising temperature, the mean development times reported by Calado (2002) [1]—19.09 (±5.70), 13.10 (±8.37), and 10.44 (±6.18) days—are close to those observed in this study: 21.9 (±1.95), 14.6 (±1.63), and 11.6 (±1.81) days, though with less variation in our results. The author also noted that females developed more slowly than males, with differences of approximately one to four days depending on temperature.

Beserra et al. (2006) [31] evaluated *Ae. aegypti* populations at temperatures of 18 °C, 22 °C, 26 °C, 30 °C, and 34 °C, observing that egg hatching did not occur at 18 °C in three of the five populations studied. However, hatching occurred at 22 °C and above in all populations, indicating that thermal tolerance can vary among populations. This could explain the absence of *Ae. terrens* egg hatching at 15 °C in our study, suggesting species-specific thermal adaptations. Regarding development time, all five populations of *Ae. aegypti* exhibited a decrease in development duration as temperature increased [31]. The authors also assessed *Ae. aegypti* at 18 °C, 22 °C, 26 °C, 28 °C, 32 °C, and 34 °C across three different populations. The development pattern observed followed a general trend for the genus, with the shortest development times recorded at 34 °C. The highest egg viability occurred at 28 °C in two populations and at 26 °C in one population, temperatures that are close to the 27 °C optimal egg hatching temperature [3] identified for *Ae. terrens* in the present study.

## 5. Conclusions

Our study on the influence of temperature on *Ae*. *terrens* development provides three key insights based on the results obtained. First, each developmental stage of the species exhibits distinct sensitivity to temperature, with sensitivity generally increasing as development progresses. The quadratic temperature term proved significant for most stages analyzed, supporting the hypothesis that each stage has an optimal temperature, likely close to those tested in this study. Additionally, small differences in environmental response, as well as sex-based differences, can become more pronounced throughout the development process.

## Figures and Tables

**Table 1:** Hatching rate percentage and predicted values for *Aedes* (*Protomacleaya*) *terrens* eggs at 15 °C, 20 °C, 25 °C, and 30 °C under laboratory conditions.

Temperature	Eggs	Hatching Rate	CI 95%
15	214	0.00	0.00–0.00
20	181	0.05	0.03–0.09
25	226	0.37	0.32–0.42
30	236	0.29	0.24–0.34

**Table 2:** Mean stage durations, minimum and maximum total development times of *Aedes* (*Protomacleaya*) *terrens* at 20 °C, 25 °C, and 30 °C under laboratory conditions.

Temperature (°C)	L1–L2 (SD)	L2–L3 (SD)	L3–L4 (SD)	L4–Pupa (SD)	Pupa–Adult (SD)	L1–Adult (SD)	Total Development (Days)	
							Min.	Max.
20	4.43 (0.98)	2.43 (0.53)	3.43 (1.13)	6.86 (1.07)	4.71 (1.95)	21.9 (1.95)	20	25
25	3.71 (0.99)	1.97 (0.84)	2.34 (1.03)	3.79 (1.21)	2.79 (0.89)	14.6 (1.63)	12	22
30	2.30 (0.84)	1.99 (0.75)	1.89 (0.89)	3.21 (1.33)	2.24 (0.78)	11.6 (1.81)	8	15

**Table 3:** Comparison between different linear models predicting the logarithm of the number of days (log(Days)) based on the variables Sex, Temperature, and Temperature^2^. The model that is most plausible, according to the Akaike Information Criterion (AIC), is indicated in bold.

Model	df	AICc	∆AICc	Weight
**log(Days)~Sex + Temperature + Temperature^2^**	**5**	**−308.8**	**0.00**	**0.759**
log(Days)~Temperature + Temperature^2^	4	−306.5	2.30	0.241
log(Days)~Sex + Temperature	4	−294.5	14.30	0.001
log(Days)~Temperature	3	−292.7	16.02	0.000
log(Days)~Sex + Temperature^2^	4	−286.3	22.49	0.000
log(Days)~Temperature^2^	3	−284.7	24.10	0.000
log(Days)~Sex	3	−117.4	191.35	0.000
log(Days)~1 (null model)	2	−114.1	194.65	0.000

The model that is most plausible, according to the Akaike Information Criterion (AIC), is indicated in bold.

## Data Availability

The data of the current study are available from the corresponding author upon reasonable request.

## Supplementary Materials

The following supporting information can be downloaded at: https://www.mdpi.com/article/10.3390/life15071038/s1, Table S1. Effect of the variables Temperature, Temperature^2^, and Sex on the development of each ontogenetic stage. The model structure follows the most plausible model identified for development up to the adult stage of *Ae.* (*Protomacleaya*) *terrens*. Each model was constructed as Stages/Model~Temperature + Temperature^2^ + Sex. The columns, from left to right, show: each model/stage analyzed, the contribution of each variable, including the coefficient (effect size; β), standard error (SE), 95% confidence interval (lower and upper bounds), and *p*-value. The values presented correspond to those shown in Figure 4 of the manuscript. Figure S1—Validation of assumptions for the best model estimating the egg hatching of *Ae.* (*Protomacleaya*) *terrens*. Figure S2—Validation of assumptions for the best model estimating larval development time until the adult stage of *Ae.* (*Protomacleaya*) *terrens*. Assumption tests included, from left to right: QQ plot of theoretical vs. empirical residuals and deviation comparison via Levene’s homogeneity test and uniformity between groups. Figure S3—Histogram showing the distribution of residuals from the model estimating larval development time to the adult stage of *Ae.* (*Protomacleaya*) *terrens*.
