# Supplementary material for: Impact of Thermal Variation on Egg Hatching and the Life Cycle of Aedes (Protomacleaya) terrens (Diptera: Culicidae) in a Laboratory Environment
Source: Life (Basel). 2025 Jun 30;15(7):1038. doi: 10.3390/life15071038 (PMC12299280; doi:10.3390/life15071038)
Supplement: Supplementary file 1 [file life-15-01038-s001.zip › life-3657186-supplementary.pdf]

SUPPLEMENTARY MATERIAL

Table S1. Effect of the variables Temperature, Temperature², and Sex on the development of each ontogenetic stage. The model structure follows the most plausible model identified for development up to the adult stage of *Ae.* (*Protomacleaya*) *terrens*. Each model was constructed as Stages/Model ~ Temperature + Temperature² + Sex. The columns, from left to right, show: each model/stage analyzed, the contribution of each variable, including the coefficient (effect size; β), standard error (SE), 95% confidence interval (lower and upper bounds), and p-value. The values presented correspond to those shown in Figure 4 of the manuscript.

Stages/Model	Variable	Coefficient	SE	CI_low	CI_high	p-value
L1–L2	Temperature	0.40	0.37	-0.33	1.12	0.28
	Temperature²	-0.01	0.01	-0.03	0.00	0.09
	Sex	-0.12	0.11	-0.34	0.11	0.31
L2–L3	Temperature	-0.63	0.47	-1.54	0.28	0.18
	Temperature²	0.01	0.01	-0.01	0.03	0.19
	Sex	-0.26	0.14	-0.54	0.02	0.07
L3–L4	Temperature	-0.78	0.45	-1.66	0.10	0.08
	Temperature²	0.01	0.01	0.00	0.03	0.13
	Sex	0.05	0.14	-0.22	0.32	0.70
L4–Pupa	Temperature	-2.17	0.42	-2.98	-1.35	0.00
	Temperature²	0.04	0.01	0.02	0.05	0.00
	Sex	-0.09	0.13	-0.34	0.16	0.50
Pupa–Adult	Temperature	-1.91	0.39	-2.68	-1.14	0.00
	Temperature²	0.03	0.01	0.02	0.05	0.00
	Sex	0.00	0.12	-0.24	0.24	1.00

Tables and figures representing the model validation are presented below.

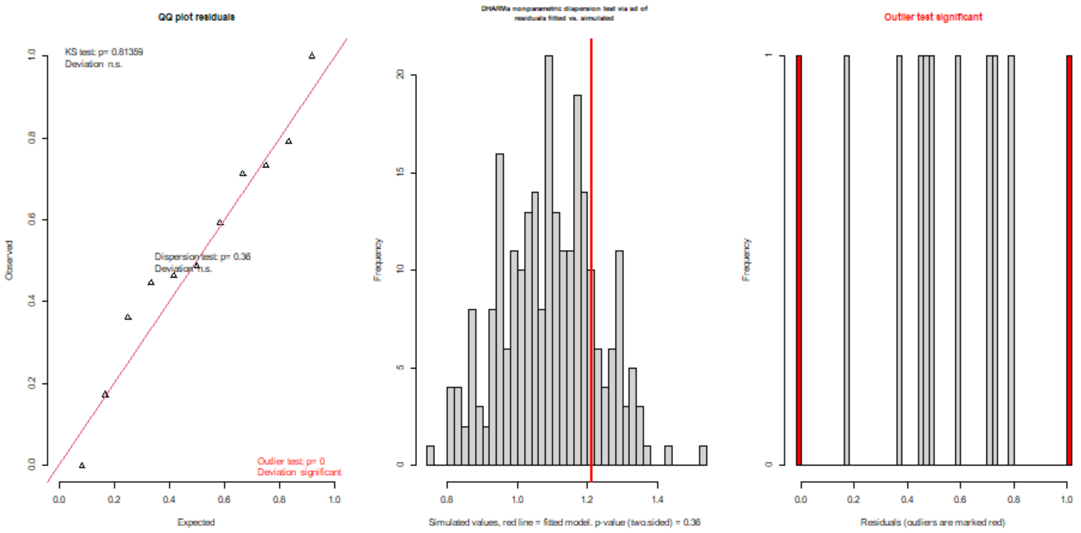

Figure S1 – Validation of assumptions for the best model estimating the egg hatching of *Ae.* (*Protomacleaya*) *terrens*.

Assumption tests included, from left to right: QQ plot of theoretical vs. empirical residuals; residual histogram for the non-parametric DHARMa dispersion test comparing observed and simulated data; and residual outlier histogram, with red columns indicating significant values. Overall, despite the model fitting reasonably well, issues with outliers were observed. The detection of outliers is likely due to the low number of replicates for the 30 °C hatching experiment, which is expected in such cases. Therefore, although the model did not fully meet all assumptions, it presented sufficient robustness to proceed with the analysis.

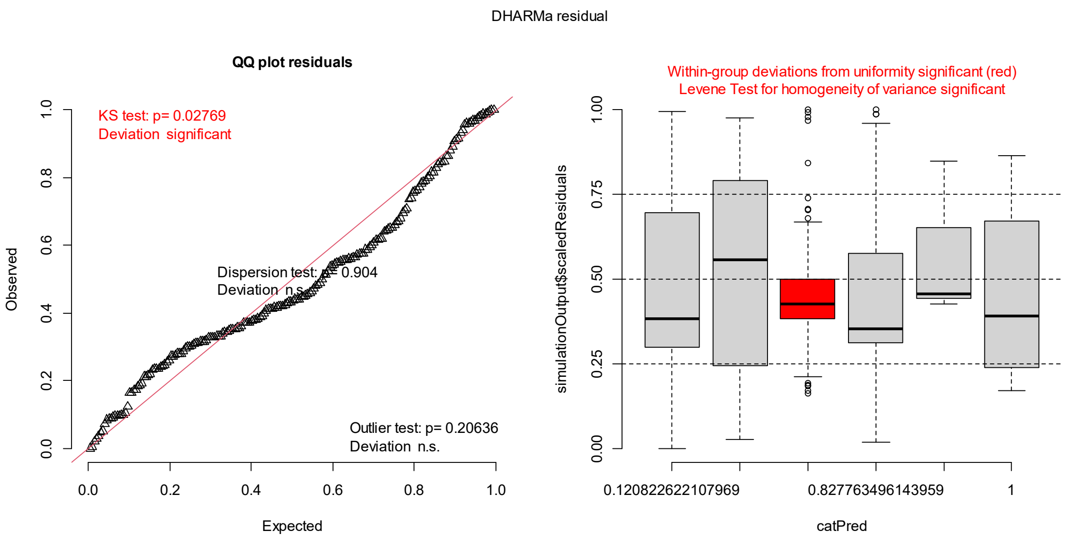

Figure S2 – Validation of assumptions for the best model estimating larval development time until the adult stage of *Ae.* (*Protomacleaya*) *terrens*. Assumption tests included, from left to right: QQ plot of theoretical vs. empirical residuals and deviation comparison via Levene's homogeneity test and uniformity between groups.

Red values indicate if any assumptions (outlier presence, dispersion, or uniformity) were not satisfied. Although the model did not fully meet all assumptions, it showed robust adjustment with residuals close to zero, and the high variability in development times may explain the deviation from normality (see Figure S3).

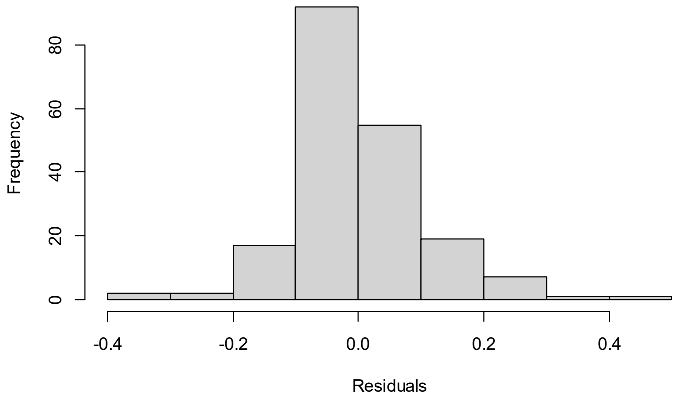

Figure S3 – Histogram showing the distribution of residuals from the model estimating larval development time to the adult stage of *Ae.* (*Protomacleaya*) *terrens*.

The distribution does not follow a normal pattern (Shapiro-Wilk test, $W = 0.9$, p-value < 0.001).

The best model selected passed the standard validation procedure for residual normality (Kolmogorov-Smirnov test, $D = 0.179$, $p = 0.814$) and dispersion (dispersion test = 1.111, $p = 0.36$) but did not pass the outlier test (2 out of 11 observations, $p < 0.001$), which is expected given the small sample size.
